# Host Transcriptional Response to Ebola Virus Infection

**DOI:** 10.3390/vaccines5030030

**Published:** 2017-09-20

**Authors:** Emily Speranza, John H Connor

**Affiliations:** Department of Microbiology, Bioinformatics Program, National Emerging Infectious Disease Laboratories, Boston University, Boston, MA 02118, USA; speranza@bu.edu

**Keywords:** Ebola virus disease, host response, transcriptomic, RNA-Seq, microarray, immune response

## Abstract

Ebola virus disease (EVD) is a serious illness that causes severe disease in humans and non-human primates (NHPs) and has mortality rates up to 90%. EVD is caused by the *Ebolavirus* and currently there are no licensed therapeutics or vaccines to treat EVD. Due to its high mortality rates and potential as a bioterrorist weapon, a better understanding of the disease is of high priority. Multiparametric analysis techniques allow for a more complete understanding of a disease and the host response. Analysis of RNA species present in a sample can lead to a greater understanding of activation or suppression of different states of the immune response. Transcriptomic analyses such as microarrays and RNA-Sequencing (RNA-Seq) have been important tools to better understand the global gene expression response to EVD. In this review, we outline the current knowledge gained by transcriptomic analysis of EVD.

## 1. Ebola Virus Disease

*Ebolavirus* is a member of the *Filoviridae* family of negative-sense single-stranded RNA viruses. *Ebolaviruses* are the causative agents of Ebola virus disease (EVD), which is a serious illness that can cause up to 90% case fatalities in humans and non-human primates (NHPs) [1]. Currently, there are no licensed vaccines or treatments for EVD. There are five species of the genus *Ebolavirus*: Zaire (EBOV), Sudan (SUDV), Bundibugyo (BDBV), Tai Forest (TAFV), and Reston (RESTV). Four of the five *Ebolavirus* species are known to cause disease in humans (EBOV, SUDV, BDBV, and TAFV) with three of them linked to sporadic outbreaks in Central and Western Africa (EBOV, SUDV, and BDBV) [2]. The recent outbreak in West Africa in 2013–2016, the largest outbreak to date, was caused by the Makona variant of EBOV. This outbreak resulted in ~28,600 cases and ~11,000 deaths [3], emphasizing the need to better understand EBOV pathogenesis.

*Ebolavirus* infection can be initiated by virus entry into the body through mucosal sites or by direct contact (through open wounds). Once inside the body, the primary target cells for *Ebolavirus* are thought to be dendritic cells and macrophages which become actively infected [4,5]. These infected cells then carry the virus to the primary organs of viral replication, which include the liver, spleen, and lymph nodes [1]. Here, the virus undergoes significant replication and expansion. During this expansion an infected individual will begin to present with early disease symptoms.

Symptoms of EVD usually start with a prodromal period of general malaise and fever. This can initiate 2–21 days after initial exposure to the virus. As the disease progresses, additional symptoms such as nausea and vomiting, loss of appetite, and severe diarrhea leading to dehydration develop [2]. People infected with *Ebolavirus* can progress to a hemorrhagic fever, eventually presenting with disseminated intravascular coagulation (DIC), multi-system organ failure, and death. The presentation of DIC is often coupled with increased liver enzymes such as alanine aminotransferase (ALT) and aspartate aminotransferase (AST), signifying poor liver health and which are associated with poor disease outcome [6].

EVD pathogenesis is linked to the overactivation of innate inflammatory signaling and the absence of dendritic cell activation. The mechanism in which this excessively exuberant response is triggered is unknown. Macrophages, but not dendritic cells [7], are activated upon infection with *Ebolavirus* and human macrophages have been shown to produce many innate immune signals such as interferons [8]. The lack of dendritic cell activation may hinder the ability of the host to mount a productive adaptive immune response. Lack of dendritic cell activation coupled with an increase in checkpoint markers such as CTLA-4 (cytotoxic T-lymphocyte-associated protein 4) and PD-1 (programmed cell death 1) [9] on the surface of T-cells during infection are thought to be contributing factors associated with lymphocyte death during infection. Lymphocyte death has also been noted in both mouse [10] and non-human primate (NHP) [11] models of infection.

Significant focus has been put on understanding the innate immune response to EVD. The innate immune response to EVD contains two main areas of study: the antagonism of the interferon response and the cytokine response. Many pro-inflammatory cytokines have been shown to be associated with EVD. IL-10 and IL-8 levels have been shown to increase in human fatal infections of SUDV [12] and EBOV [13]. IL-6 (another pro-inflammatory cytokine) has conflicting reports as one study found it to be high in EBOV survivors [13] and another study found IL-6 to be higher in fatal EBOV cases [12]. A characteristic feature of EVD in humans is a drastic increase in inflammatory cytokines that occurs during the symptomatic phase of the infection. This has been observed in many NHP models of infection [14,15] and has been suggested to be similar to the response seen in patients experiencing sepsis and severe viral diseases such as Marburg fever [16] or severe influenza infections [17].

Another aspect of the innate immune response that is of focus with regards to *Ebolavirus* infections is the antagonism of the type I interferon (IFN) response. IFN antagonism is achieved through the inhibition of intracellular recognition of pathogen-associated molecular patterns (PAMPs) by the *Ebolavirus* protein VP35 [18,19]. However, recent findings suggest that VP35 inhibition of PAMPS may be cell-type specific [20]. Another *Ebolavirus* protein, VP24, has also been implicated in antagonizing the IFN response. Interferon antagonism by VP24 occurs by inhibiting phosphorylation of STAT1/2 into pSTAT1/2 and thus preventing its nuclear translocation [21]. The antagonist actions of the virus against the interferon response are thought to contribute to pathogenesis.

Many aspects of virus–host interaction can lead to different responses in infected cells and therefore lead to the initiation of different extracellular signaling events. How innate (IFN and cytokine) responses are influenced by virus infection is a challenging question to answer using a few selected genes of interest. Therefore, multiplexed technologies such as transcriptomics are attractive way to interrogate the host response to EBOV infection. Over the past decade, this approach has been used with increasing sophistication to study how EBOV infection alters the host transcriptome at the cellular and organismal level. This review addresses the current state of the field, with a focus on noting the important findings and challenging questions highlighted by this line of experimentation.

## 2. Transcriptional Response to Ebola Virus Disease In Vitro

Understanding the impact of EBOV infection on the host transcriptional response can be carried out with multiple approaches. The most direct is the use of immortalized cells or purified populations of primary cells that are infected in vitro. Infection of these cells can be done under tightly controlled conditions and the virus titer can be accurately determined.

### 2.1. Studying the Transcriptional Response in Immortalized Cell Lines

The use of immortalized cell lines to study the host response to EBOV infections provides ease of access to cells and allows for large number of replicates to be analyzed with minimal increase to cost. The transcriptional response to EBOV infection has often been studied in immortalized human liver carcinoma cell lines (HepG2 and Huh7 cells). The liver is an initial organ of EBOV replication. Thus, the liver likely plays an important role in EBOV pathogenesis, making the host liver response a key question in understanding viral dysregulation of host signaling [22]. Following initial studies in liver cells, other studies have examined the host transcriptional response in cell lines that are representative of other sites of EBOV infection such as kidney, lung tissue, and immortalized monocytes.

The earliest transcriptomic studies supported an existing hypothesis of the EBOV–host interaction, namely that EBOV is a strong suppressor of innate immune responses [19,23,24]. Infections performed in Huh7 cells [25] showed that EBOV infection resulted in only small changes in host gene expression compared to controls. Importantly, this study analyzed infection of an additional related *Ebolavirus*, RESTV, that has yet to be associated with severe disease in humans. In RESTV-infected cells, the authors observed an induction of host antiviral genes such as interferon stimulated genes (ISGs) and inflammatory cytokines. These results are consistent with the conclusion that EBOV dominantly suppresses host signaling through the action of antiviral proteins VP35 and VP24, while non-pathogenic viruses fail to suppress these antiviral responses. This negative correlation of pathogenicity versus induction of ISGs has also been observed in other viral infections, particularly influenza [26,27].

The lack of a strong innate immune response following EBOV infection was also seen following infection of HepG2 cells. The transcriptional profiles of cells infected with EBOV show little induction of an innate immune response [28,29,30]. Importantly, this lack of induction of innate immune response appears to be through active suppression of antiviral signaling in these cells by EBOV. This can be demonstrated by adding a mutation to the VP35 protein in EBOV that inhibits VP35s ability to suppresses IRF3 signaling [31]. Cells infected with only the VP35 mutant EBOV were no longer transcriptionally silent and upregulated both ISGs and cytokines compared to cells infected with wild type EBOV. This suggests that EBOV infection would normally trigger a robust innate immune response in liver cells, but that the deployment of viral proteins such as VP35 suppresses these host responses.

**Figure 1:**
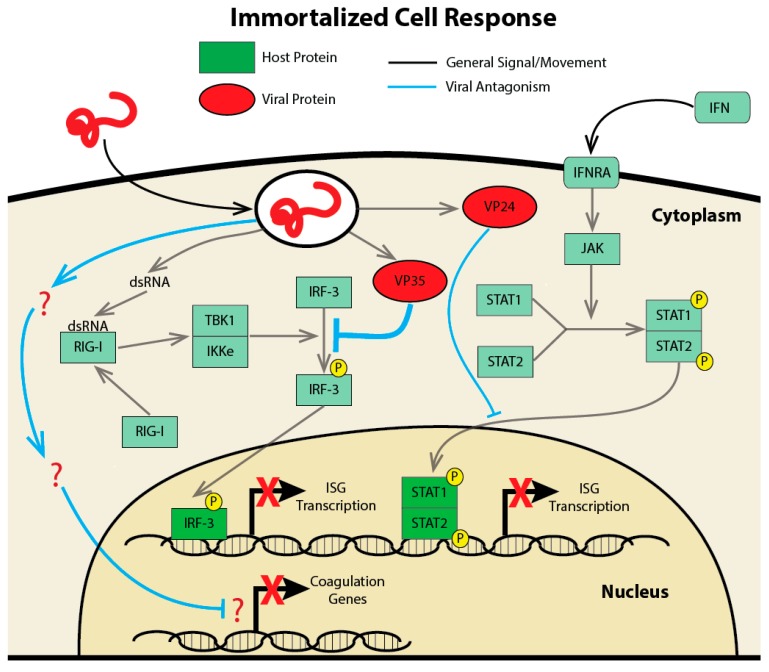
Suppression of the transcriptional response to EBOV infection in immortalized cell lines. Illustration depicts the currently understood mechanism of virus–host antagonism following EBOV entry into the cell. Upon viral uncoating, the virus releases two important viral proteins, VP35 and VP24 (represented in red). VP35 is capable of blocking activation of and signaling through the RIG-I pattern receptor, maintaining inhibitor of nuclear factor kappa B kinase subunit epsilon (IKKE)/TANK Binding Kinase 1 (TBK1) in an inactive state (host proteins are illustrated in green, with activating phosphorylation events depicted in yellow). This prevents the phosphorylation and activation of interferon response factor 3 (IRF3) and thereby the transcription of interferon (IFN) beta and IRF3-responsive interferon stimulated genes (ISGs). VP24 acts downstream of signal transducer and activator of transcription (STAT) protein phosphorylation by janus kinase (JAK) to inhibit the translocation of STATs to the nucleus, thereby inhibiting interferon signaling. Mutations in VP35 or VP24 compromise the host antagonism of each protein and result in the activation of ISG transcription following EBOV infection. Depicted in blue is the inhibition of coagulation genes that is seen following EBOV infection. Question marks signify that the mechanism by which this suppression occurs is unknown.

The suppression of innate immune signaling following EBOV infection is outlined in Figure 1 and is a common feature in immortalized cells. Analysis of the transcriptional response of THP-1 monocytes [18], immortalized lung cells (A549) [32], and immortalized kidney cells (293T) [29] following EBOV infection, all found transcriptionally silent ISGs and showed very little evidence of an innate immune response. Infection in the lungs and kidneys are secondary sites of EBOV infection, which should be considered when interpreting their results. It was also shown again that this inhibition is likely from a strong contribution of VP35 with less involvement of VP24 [18]. The suppression of innate immune responses is found across different variants of EBOV, including the Ecran (Mayinga) variant isolated in 1976 during the first EBOV outbreak, to the recent Makona variant which was the causative agent of the West Africa outbreak [32]. Interestingly, this inhibition of the IFN response is not universal across all cell types. When a different kidney cell line (769p) was infected with EBOV, there was variable inhibition of the IFN response and induction of some ISGs [29]. Similarly, when determining if retinal pigment epithelial cells, a possible location for viral persistence after recovery, could sustain EBOV infection using ARPE-19 cells, a large induction of the IFN response was evident [33]. This suggests that the ability of the virus to inhibit the interferon response likely displays cell-type-specific activity.

Another finding that has been validated across multiple transcriptomic analyses is the decrease in the transcription of genes associated with coagulation and acute phase response in cell line infections with EBOV [25,28,32]. This observation is opposite to the response seen in vivo. In vivo, EBOV is known to cause coagulation dysfunction leading to symptoms of DIC. Many pathways associated with the acute phase response are activated following EBOV infection in vivo [12,34,35]. This is accompanied by a large increase in coagulation-associated mRNAs [4]. While some cell lines show a decrease in coagulation and acute phase response genes, this is not uniformly observed across all cell lines and EBOV variants. Infection of A549 cells with the EBOV variant Makona increased the acute phase response when compared to uninfected controls [32]. Further investigation of the transcriptional response to host protein production would further help understand these differences and their relation to EVD.

### 2.2. Transcriptional Analysis of a Potential Reservoir

Bats are a hypothesized reservoir for EBOV, based both on serosurveillance studies that identified anti-EBOV antibodies in adult bats [36] and on the isolation of a closely related virus, Marburg virus (MARV), from bats [37]. However, to date no infectious EBOV has been isolated from bats. To investigate if bats have a different transcriptional response to EBOV infections, two studies carried out RNA-Seq analysis on immortalized bat cell lines [28,29]. Upon infection with wild-type EBOV, there was some evidence of an interferon response in one study [28] whereas there was no detectable interferon response in the other [29]. Infection of different immortalized bat cell lines with only VP35 mutant EBOV, which cannot block the interferon response, found an increase in the expression of many ISGs. It was also found that wtEBOV triggered a large down-regulation of many genes that was reversed when bat cells were infected with only the mutant EBOV with a VP24 that was unable to inhibit the IFN response [29]. Together these studies show that the ability of EBOV to inhibit the innate immune response is not specific to human immortalized cells.

### 2.3. Studying the Transcriptoinal Response in Primary Cells

**Figure 2:**
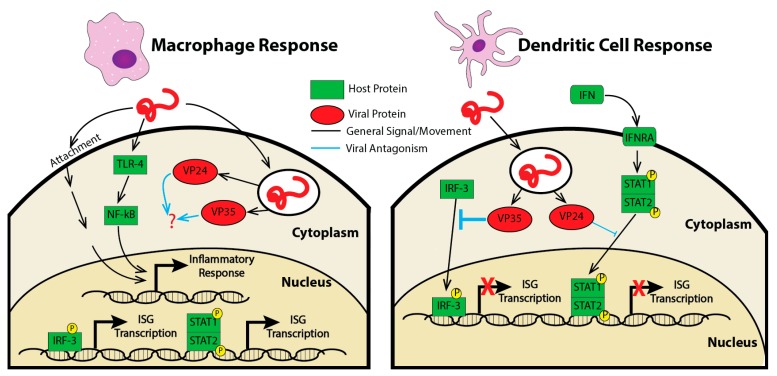
Virus–host antagonism following EBOV infection of macrophages or dendritic cells. Left panel illustrates the response to EBOV entry and attachment in macrophages. Upon attachment, activation of tool like receptor 4 (TLR-4) and nuclear factor kappa B (NF-κB) by the entry process lead to the induction of inflammatory transcripts. IRF3/IFN-induced genes are also induced in macrophage-infected cells, suggesting that the function of VP24 and VP35 as viral antagonists of these signaling pathways is compromised or that other signaling pathways are activated. The right-hand panel illustrates the interaction of EBOV and the host-response in dendritic cells. Similar to what is seen in many immortalized cell lines, VP35 will act as a strong inhibitor of IRF-3 phosphorylation blocking interferon and ISG transcription. Also, VP24 will act as an inhibitor of translocation of pSTAT1/2 to the nucleus further blocking interferon transcription.

The transcriptional response following EBOV-infection has also been studied in primary cells. Studies with primary cells are somewhat limited, as the isolation of primary cells from primary tissue targets such as the spleen and liver is challenging. This has led to a focus on cells that can be present in the circulating immune system, and on dendritic cells and macrophages, which are thought to be the initial targets of productive EBOV infection [8] and have been shown to have variable activation upon infection with EBOV [7,20]. To investigate the activation status of human dendritic cells, one study analyzed the global response of EBOV infection in human monocyte-derived dendritic cells (mdDC) (Figure 2) [38]. Consistent with what was observed in the immortalized cell lines [25,30], EBOV infection of mdDCs did not induce the transcription of innate immune response genes. This lack of an innate immune response again appears to be due to a suppression of antiviral signaling pathways by viral proteins, mostly through the effects of VP35 with minimal involvement by VP24. The same was seen with cytokine expression. This suggests that EBOV infection would induce the maturation and expression of cytokines and ISGs in human dendritic cells if it were not for the suppressive actions of VP35 and VP24. More work looking at the host response in other types of dendritic cells (DCs) such as primary DCs, tissue-specific DCs, and immature DCs would help determine if this response is mirrored in DCs or is primarily observed in mdDCs. Also, further work is needed to determine the effects of these suppressed DCs on bystander cells.

In contrast, primary macrophages show a robust host response following EBOV infection (Figure 2). Studies of early events in macrophage responses to EBOV showed that attachment of EBOV or EBOV virus-like particles (EBOV-VLPs) to primary macrophages induced changes in gene expression as early as one hour post-infection [39]. Gene expression changes persisted through to six hours post-infection. These changes in expression were generally associated with the inflammatory response. In monocyte derived macrophages (MDMs), EBOV infection in vitro followed over a longer timeframe resulted in dramatic changes in viral gene expression [40]. EBOV infection was followed by large changes in the expression of genes associated with IFN signaling, interferon stimulated genes, cytokines, and antigen presentation [40]. These data support the hypothesis that macrophages, unlike dendritic cells, are activated following EBOV infection. Of note, RESTV infection did not induce changes in gene expression in MDMs [40]. This is the opposite of what was observed in immortalized liver cells, suggesting a complex interplay of different viruses with immune response induction and raising the question of how different host responses in different infected cells contribute to the overall host response seen in vivo.

## 3. Transcriptional Response to Ebola Virus Disease In Vivo

Studying the transcriptional response to *Ebolavirus* in vivo provides an opportunity to better understand how the multitude of cell types and organ systems interact and respond to the virus as a whole. The main animal models that are used for studying the transcriptional host response to EBOV to date have been rodents and non-human primates (NHPs).

### 3.1. Rodent Models and Transcriptional Response

Mice have been used to study EVD pathogenesis for more than two decades. Since standard inbred mouse strains are not susceptible to *Ebolavirus* infection [41], three distinct virus or host modification approaches have been used to facilitate this work, each of which has its own advantages and limitations. EBOV that has been serially passaged such that it has mutated in a manner that it is pathogenic in mice (mouse-adapted) has been used to study infection in an inbred mouse with an intact immune response [41]. Studies of non-adapted virus can be carried out in mice deficient for innate immune responses [42,43]. A more recently-adopted approach uses mice with a transplanted human immune system, which allows the use of an immunocompetent system and non-adapted EBOV [44,45]. Of these approaches, the mouse-adapted (MA-EBOV) model has been used most extensively to study the host response to EBOV infection. The pathogenesis of the MA-EBOV model does not recapitulate some clinical signs seen in the human disease as there are no signs of overt hemorrhage or coagulopathy, though the mice do experience excessive weight loss, elevated temperature, and loss of appetite [46]. The later clinical signs are general to pathogenic infections in mice that are susceptible to infection. Yet the ability to easily alter the genetics of mice make them an attractive model to study the mechanisms of disease.

**Figure 3:**
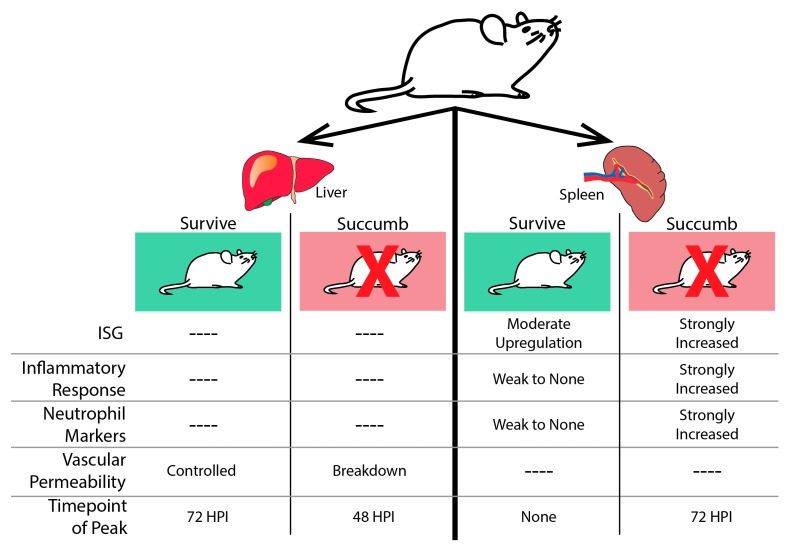
The host transcriptional response to EBOV infection in mouse liver and spleen. In the liver (left), all mice challenged with EBOV show transcriptional changes. Animals that succumb experience a peak point of transcriptional activity at 48 h post-infection (HPI.). Genes are enriched for transcripts associated with loss of vascular permeability. Surviving mice do not experience a peak of transcriptional activity until 72 HPI and do not induce genes associated with the breakdown of the vascular layers. In the spleen (right), all animals challenged with EBOV mount a transcriptional response to infection. In animals that will succumb to disease, peak transcriptional activity is observed at 72 HPI. These transcripts are associated with a strong induction of ISGs, the inflammatory response, and evidence of neutrophil infiltration. In animals that survive, there are lower levels of transcriptional activity throughout infection. There is moderate up-regulation of ISGs and a weak inflammatory response.

Infection of mice with both pathogenic and non-pathogenic EBOV has indicated that there are strong transcriptional responses to EBOV infection in tissues and blood. Studies of the transcriptional response in the spleen of EBOV-infected mice have most effectively demonstrated that there are two distinct responses to infection based on pathogenicity. One study compared five different virus infections: EBOV-MA and EBOV-NP_ma_/VP24_ma_, which are pathogenic in mice and wtEBOV, EBOV-NP_ma_, or EBOV-VP24_ma_ which are non-pathogenic in mice [47]. Following either pathogenic or non-pathogenic challenge, all mice showed an up-regulation of transcripts involved in the innate immune response and inflammation by 72 h post-infection, including a strong upregulation of ISGs [48]. Following this conserved host response to all forms of EBOV challenge, there were stark differences observed as infection progressed. In animals that succumbed to infection, there were a greater number of differentially regulated genes seen at the 72 h time-point compared to animals that survived challenge (Figure 3) [47]. Upregulated genes were mostly associated with leukocyte infiltration and degranulation suggesting an increase in neutrophils late in infection. This is a logical finding, as neutrophilia has been previously described in humans [49] and macaque models of infection at the late stages of disease [50].

Similar results have been seen in experiments using genetically diverse strains of mice derived from the collaborative cross (CC) [51,52]. Through infection of CC mice with MA-EBOV using this genetically diverse background, susceptible versus non-susceptible mouse strains could be identified. Gene expression changes analyzed in the spleen found similar results to those described above [53,54] where susceptible mice saw a large number of differentially expressed genes at the late stages of infection and non-susceptible mice had a very low level of differentially-expressed genes at this time. In the livers of these mice, both susceptible mice and non-susceptible mice experienced a large number of differentially-regulated genes (Figure 3). However, the timing of gene expression was offset, with susceptible mice showing an earlier onset of differentially-regulated genes. Functional analysis of differentially-expressed genes in animals that succumbed to MA-EBOV challenge suggested that susceptibility was related to an increase in vascular permeability.

The existence of a robust antiviral host response centered on IFN is also supported by experiments using wtEBOV infection in mice lacking intact antiviral signaling molecules [42,55]. Consistent with earlier studies [51,53,54], these studies have found that in spleens of wild-type mice, wtEBOV and MA-EBOV infection resulted in a marked induction of genes associated with inflammation and the IFN response. When antiviral signaling is impaired through the removal of the mitochondrial antiviral-signaling protein (MAVS), an important component of the IFN induction pathway, the IFN response is blunted and there are very few transcriptional changes of IFN-stimulated genes [42]. Another key finding was the discovery natural killer (NK) cell transcriptional signatures present in mice that survived infection. NK cells have been of interest because of their association with survival of EBOV infections [56,57], thus supporting the transcriptional signature.

Mouse models, such as humanized mice, which show promise to better parse out the dynamics of the host response to infection, have not had transcriptional analysis performed as of yet. Humanized mice display clinical signs that more closely relate to human disease [44,45] and are useful for understanding mechanisms of disease progression and pathogenesis [5]. This model could provide more insight into detailed pathogenesis and host response during an EBOV infection, transcriptionally.

### 3.2. Non-Human Primate Models and Transcriptional Response

The most widely-accepted animal model of EVD for the study of countermeasures such as vaccines and therapies is the non-human primate (NHP) model [46]. NHPs display almost all of the clinical signs of acute EVD, including DIC and hemorrhage. Despite most NHP survivors needing experimental treatments to survive infection, individuals who survive challenge or have an extended course of disease can show neurologic or ophthalmologic complications [58,59] similar to human cases [60,61]. Similar to studies in mice, studies of EVD in NHPs allow the collection of samples multiple times over the course of infection from each challenged subject, providing information on the overall course of infection as well as variation between individuals. Currently, the host transcriptional response to EVD has been studied in two NHP models, rhesus (*Macaca mulatta*) and cynomolgus (*Macaca fascicularis*) monkeys, supported by the annotation of the rhesus and cynomolgus transcriptomes [62,63,64]. An advantage of the NHP system is that the response of the immune cells circulating in the blood following EBOV exposure has been extensively characterized, allowing comparisons to human transcriptome datasets, which is not currently possible with other animal model comparisons (see below). Since the response seen in both rhesus and cynomolgus NHPs is extremely similar, we will discuss results from the two different models simultaneously. Also, two main routes of infection have been used to study the host response to infection: intramuscular injection and aerosol exposure. Again, the differences in these two responses are very similar and will be combined in the following description.

**Figure 4:**
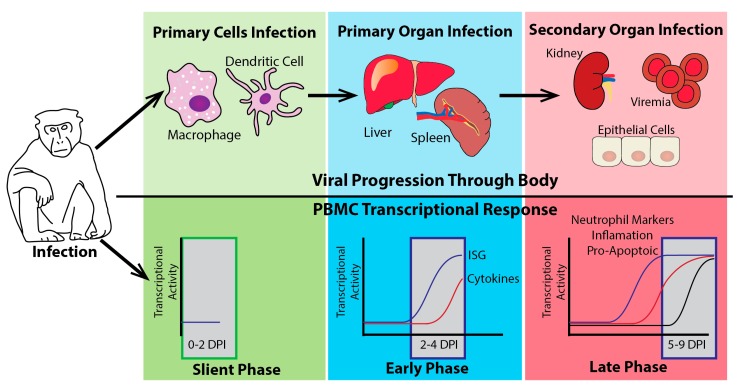
The host transcriptional response to EBOV challenge in NHPs. The illustration shows three distinct stages of transcriptional activation seen in peripheral blood mononuclear cells (PBMCs) following EBOV infection of NHPs. The top half (above the black line) shows the likely viral progression of EBOV from the infection of initial target cells (green phase) to replication in the spleen and liver (blue phase) and finally dissemination into secondary organs (red phase). The bottom panels illustrate the transcriptional activity of different gene families during this progression. The earliest phase of transcriptional activity (0–2 days post-infection (DPI)), in the green box, is the silent phase where very little if any transcriptional activity is present in the PBMCs. During this phase, the virus has initiated infection in primary cell targets. As the virus moves to its primary organs of infection, a robust early phase of transcriptional activity is observed (2–4 DPI). Most notable in this phase is the increase in ISGs. This ISG induction is closely followed by the induction of cytokine genes. Finally, as the virus begins to disseminate through the whole body through the blood (viremia) and begins to infect secondary organ targets, the transcriptional activity enters the late phase characterized by the induction of many pro-inflammatory genes, pro-apoptotic markers, and neutrophil markers in PBMCs.

As a first-order approximation, the NHP circulating immune response to EBOV challenge can be separated into four separate transcriptional phases (Figure 4). Following EBOV challenge, there is an initial “silent” phase where there is little to no reproducible host response in immune cells circulating in the peripheral blood. It is likely that there is a host response at the site of infection or at a point of initial trafficking, but these sites have yet to be analyzed. Following this “silent phase” there is an early phase of host response to EVD infection. This early phase response can be detected prior to or at the time of other markers of infection such as fever or viremia [48,65]. This early phase is in direct contrast to much of the datasets from cell lines and reviews which suggested that there is a suppression or evasion of the host immune response by EBOV. In a NHP model of infection, as early as 2–4 days post-infection there is a strong up-regulation of many type-I IFN-stimulated genes [48,65,66,67]. This early innate immune response occurs to a lesser extent or is not detected in animals that are asymptomatic for disease indicating that IFN is a result of productive infection [48,65,66]. This IFN response in intramuscular infections peaks at around four DPI and is sustained to the end of disease [48]. This general and early IFN response is similar to what has been observed for many other viruses including human influenza infections [68,69], macaque influenza infections [70], as well as Lassa virus (LASV) and MARV infections in macaques [71,72,73].

The early phase of infection is closely followed by the up-regulation of many cytokine genes [48,65]. These cytokines (e.g., CCL8, FAS, and IL-6) begin to see significant expression in fatal cases starting at 4–5 days post-infection and are sustained through to the end of infection. In animals that do not succumb to infection, cytokine expression in the peripheral blood mononuclear cells (PBMCs) is drastically lower than that observed in animals that succumb to disease [48,66]. This is consistent with *Ebolavirus* infections leading to an overactivation of proinflammatory cytokines which contributes to disease pathogenesis [14]. This up-regulation of many cytokines is concurrent with NF-κB and TNF-α activation determined through network analysis of the cytokine response [65].

The later stages of infection (5–11 days post-infection) are characterized by extreme changes in gene expression in NHPs exposed to EBOV. Around 2000–3000 genes are usually found to be differentially regulated during the late stages of disease, depending on the fold-change cutoffs used [65,66,67,74]. Gene ontology classification of genes showing altered regulation at late stages of the disease found that many are associated with apoptosis [65] and immune dysfunction through increases in inflammatory pathways and decreases in T-cell activation pathways [65,66,67]. There is evidence of neutrophil marker genes appearing in PBMCs during the late stages of disease [48,74] which may suggest immature neutrophils being present in high quantities in the blood, since neutrophils are not regularly found in PBMCs. In animals that are either asymptomatic for disease or survive disease due to vaccination or experimental therapeutic treatments, this over-activation and suppression of many transcripts is seen to a much lesser extent. Activation of T- and B-cell function genes is observed, coupled with markers for natural killer cells [66]. In surviving animals, the end stage of disease is also associated with an increase in IFN gamma signaling [66]. IFN gamma has been suggested to play an important role in viral clearance and suggests a mechanism for reducing viral loads [75,76].

Further work in NHPs has been done to fully understand the mechanism of the rVSV-EBOV vaccine [77,78,79]. This vaccine platform utilizes a recombinant form of the vesicular stomatitis virus (VSV) that expresses the EBOV-glycoprotein (GP) on the surface (rVSV/ZEBOV-GP) [78] and showed approximately 100% effectivity in humans [80]. Transcriptomic analysis was performed on macaques with samples taken before administration of the vaccine, after the vaccine was given, and throughout challenge with EBOV to determine transcriptionally which important components of the immune response contributed to protection [67,81]. Following exposure to the vaccine, around 100 transcripts were found to be differentially regulated. These transcripts are mostly associated with TLR signaling and some innate immune genes [81]. After challenge with EBOV, these animals experience low levels of circulating virus that are quickly cleared. These challenged animals experience an increase in some innate immune genes, including many IFN-stimulated genes [67,81], suggesting that despite being protected, these animals still had a modest immune response to challenge. Importantly, animals that were not vaccinated against EBOV saw a continued increase in the expression of many innate immune genes.

There are fewer transcriptomic analyses of tissues response to EBOV in NHPs compared to the mouse models. However, one study suggests that infected tissues have a robust host response to EBOV infection. The host transcriptional response in tissues is similar to the transcriptional response identified in PBMCs, with early expression appearing in the spleen, liver, and pancreas, concurrent expression in the adrenal gland, and delayed expression in the lymph nodes. Expression in the brain was barely detectable and was only observed in late stages of disease [48]. Additional sequencing sets in tissues would help to further understand the host transcriptional response in tissues and how it relates to the peripheral blood response.

## 4. Transcriptional Response to Ebola Virus Disease in Humans In Vivo

The analysis of the host transcriptional response to EBOV infection in NHPs has served as an important bridge for comparing and understanding the host transcriptional response of humans infected with EBOV in the 2013–2016 West African outbreak. Two recent studies have analyzed the global transcriptional response in the peripheral blood of infected patients in Guinea [82] and a longitudinal study of the response over time in a patient treated at the NIH clinical center [83].

The first of these two studies involved a patient cohort from Guinea during the outbreak that were taken at the European Mobil Lab (EMLab) [82]. The focus of this study was to analyze transcriptional differences observed in surviving patients as compared to fatalities. This analysis showed that patients that succumbed to EBOV infection had a stronger accumulation of different pathogen response pathways than those that survived infection, independent of the number of viral genome copies in the blood. These included innate immune genes such as ISGs and cytokines and genes associated with the acute phase response and liver-specific mRNAs. These changes are largely in alignment with what has been seen in the blood of NHPs infected with EBOV. The strongest signals are those involved in the innate immune response and particularly type-I IFN response [48,66,74]. Also consistent with the NHP data are the high levels of cytokine genes detected, which were stronger in fatal cases than survivors [48], consistent with what has been seen at the protein level in patients treated in the U.S. [35]. Digital cell quantification (DCQ) analysis of the human data, using the transcriptional response to predict changes in cell populations, highlighted natural killer (NK) cell density increases correlating with survival [42], a finding that is supported by mouse DCQ analysis of EBOV infection [42]. NK cells have been shown to be increased in human survivors through FACS analysis [57]. Importantly, there was no increase in neutrophil markers in humans infected with EBOV, in sharp contrast with data obtained in NHPs.

The findings of this large patient cohort study are in general agreement with a second study of a single patient treated at the NIH Clinical Center [83], whose transcriptome was followed almost daily over the post-admission disease course. This dataset showed that the infected individual had an early response to EBOV infection that was dominated by high levels of innate immune genes, including type-I ISGs and cytokines, similar to NHP data and the other human study. This innate immune response began to subside after a significant treatment course, correlating with a decreasing viral load and an increase in adaptive immune gene signatures. The increase in adaptive immune gene subsets is in line with FACS analysis showing an increase in B-cell subtypes in patients treated in the U.S. following EBOV infection [84]. Interestingly, this patient had neutrophil markers present in the circulating blood long after viral clearance.

In addition to studies focusing on the mRNA coding transcriptome, there have also been studies that have focused the non-coding transcripts present in blood following EBOV infection. A study looking at patient samples from Sierra Leone identified microRNAs (miRNAs) whose regulation is altered in relation to viral load and selected a set of miRNAs that have potential to be used as a biomarker for survival [85]. Many of the miRNAs found in human clinical samples also correlated with miRNAs discovered in NHPs, consistent with the idea that these are part of a response to EBOV infection in both humans and NHPs. This work bears future additional study to identify these and other potentially useful noncoding RNAs.

## 5. Comparison of Various Platforms

Across the various platforms used to study EVD, there are gene sets that show agreement in their general pattern of regulation across many different models of EVD (Table 1). Most notably, in almost all the different models for studying EVD, there is a large upregulation of many IFN stimulated genes. ISGs are upregulated in all models with the exception of some types if immortalized cell lines (liver cells, A549s, 293T, and THP-1) [18,19,21,23,25,28,29,30,32] and monocyte derived dendritic cells [38]. Especially notable is the fact that ISGs are strongly upregulated in response to EBOV infection in all of the animal models [47,48,53,54]. The increase in ISGs is also seen in humans [82,83] where there is an association between a larger amount of ISG expression and a worse outcome. This is especially notable since IFN-beta (IFNβ) treatment has been suggested as a possible therapeutic for EVD based on data from immortalized cells. When this approach was tested in animals, it was found to provide no strong protection against disease in NHPs [86] and a small trial in West Africa showed no significant benefit of treatment on patient outcome [87].

The limited data from human infections has identified important differences between the human transcriptional response and transcriptional response seen in animal models. One large difference is that the transcriptome from EBOV-infected patients in Guinea were found to have very high levels of acute phase genes circulating in the peripheral blood [82]. The NHP PBMC data does not show this same signature [48,74]. Furthermore, data from cell lines suggested a decrease in the acute phase response mRNAs associated with coagulation when cells were infected with pre-2014 strains [25,32] but was induced with the West Africa Makona variant [32].

Analysis of survival factors in animal models can possibly help inform treatments in humans. Though most NHP survivors have either been vaccinated for the virus or received therapeutics, and the mouse model requires attenuation of the virus to create mouse-adapted forms, many factors found in either of these models agree with what was found in humans. Most notable is the association of natural killer cells and survival in both mice [42] and humans [82]. Natural killer cells have been previously shown to be associated with protection against EBOV infections [56] as well as IFN-gamma (IFN-γ) [75] which is a byproduct of natural killer cells and CD8+ T-cells.

## 6. Conclusions and Future Directions

Transcriptional analysis of the host response to EBOV infection has greatly increased our understanding of the disease progression and pathogenesis. An important finding highlighted through transcriptomics is the strong induction of an IFN-like response following infection in vivo [48,65,66]. This response is one of the strongest and earliest responses to infection in NHPs and mice [42,47,48]. In other hemorrhagic fever virus infections it has been suggested that the IFN response precedes viremia and is the earliest response to infection [71,73]. These findings are particularly interesting given the difference of in vitro and in vivo host responses. The lack of a robust innate immune response of many cells in vitro [18,20,32,33,40] raises the question of which cells are driving the strong response in vivo. Future work analyzing which cell subsets in vivo are driving this response will help define the source of the early response to infection.

Transcriptional analysis has also led to an appreciation of the entire scope of the immune response to infection. The discovery approach enabled by microarray and next generation sequencing (NGS) have allowed the identification of cytokine induction events that had not been previously associated with disease [48]. Importantly, the scope of the upregulation of pro-inflammatory cytokines during the infection course can now be more fully appreciated. Transcriptomic analysis has also identified transcription factors that are activated or suppressed following infection [40,66,74]. Despite these strengths, the analysis of RNA species alone does not provide a complete picture of the overall host response to infection. Future work that couples transcriptomic analysis with proteomics analysis could further determine the downstream effects.

The new knowledge gained from these studies has several potential applications. One exciting opportunity is that the host response could be used as a biomarker. Work has already been performed in human samples to develop potential biomarkers for prognosis [82,85]. The host transcriptional response may also be useful as a diagnostic. Current diagnostic assays rely on the virus circulating at high levels in the peripheral blood and were not strongly effective during the West Africa outbreak until 72 h after the onset of symptoms [88]. One way to improve upon this diagnostic would be to measure the host transcriptional response. This has been done in the context of upper respiratory infections [69,89] and to successfully distinguish bacterial from viral infections [90] without the need to isolate the causative agent. Preliminary work to develop an early diagnostic for EVD has been done using in vitro data and shows initial promise for its utilization [91]. With a large dataset from humans and an expansion of available NHP datasets, development of an EBOV diagnostic based on transcriptional changes prior to the onset of symptoms is an achievable goal.

To date, there is only one available dataset with transcriptional analysis of tissues [48]. Through this dataset and in vitro datasets, there is evidence that different tissues may respond differently to EBOV infection [18,30,32,33]. Tissues of interest include liver, lymph nodes, spleen, kidney, brain, and eye. The first four are known sites of infection either during the early stage or the late stage in NHPs. The last two are interesting due to the increased prevalence of post-Ebola syndrome and EBOV persistence in immune-privileged sites [61,92,93]. This would also help to better understand the cell-type specificity of the response to EBOV infection.

## Figures and Tables

**Table 1:** Comparison of general gene transcription across the various platforms.

Gene Group	Immortalized Cells	Primary Cells	Mouse	NHP	Human
ISGs	Suppressed in most cell lines	Upregulated in Macrophages	Upregulated in spleens	Early Response Upregulated	Upregulated
Pro-inflammatory Cytokines	Mostly suppressed	Upregulated in Macrophages	Upregulated	Mid Response Upregulated	Upregulated
Coagulation	Suppressed				Upregulated
Neutrophil associated Transcripts	N/A	N/A	Upregulated in spleens	Upregulated in PBMCs	No Significant Change
Natural Killer Cell Transcripts	N/A	N/A	Upregulated in Surviving Animals	No Significant Change	Upregulated in Survivors
Acute Phase Response	Suppressed	N/A	N/A	Upregulated	Upregulated
